# A Prospective Study of Lumbar Facet Arthroplasty in the Treatment of Degenerative Spondylolisthesis and Stenosis: Early Cost-effective Assessment from the Total Posterior Spine System (TOPS™) IDE Study

**DOI:** 10.36469/001c.33035

**Published:** 2022-03-25

**Authors:** Jared D. Ament, Amir Vokshoor, Yaser Badr, Todd Lanman, Kee D. Kim, J. Patrick Johnson

**Affiliations:** 1 Cedars Sinai Medical Center, Los Angeles, California; Neuronomics LLC, Los Angeles, California; Neurosurgery & Spine Group, Los Angeles, California; Institute of Neuro Innovation, Santa Monica, California; Sierra Neuroscience Institute, Glendale, California; 2 Neuronomics LLC, Los Angeles, California; Neurosurgery & Spine Group, Los Angeles, California; Institute of Neuro Innovation, Santa Monica, California; 3 Sierra Neuroscience Institute, Glendale, California; 4 Cedars Sinai Medical Center, Los Angeles, California; 5 University of California, Davis

**Keywords:** cost-effectiveness, Total Posterior Spine System, motion preservation, lumbar spondylolisthesis, lumbar stenosis, cost analysis, decision analysis, TOPS(TM)

## Abstract

**Background:** Given the increased attention to functional improvement in spine surgery as it relates to motion preservation, activities of daily living, and cost, it is critical to fully understand the healthcare economic impact of new devices being tested in large FDA randomized controlled trials (RCT). The purpose of this analysis was to comprehensively evaluate the cost-effectiveness of the novel Total Posterior Spine (TOPS™) System investigational device compared with the trial control group, standard transforaminal lumbar interbody fusion (TLIF).

**Objective:** To evaluate the cost-effectiveness of TOPS™ compared with TLIF.

**Methods:** The study patient population was extracted from a multicenter RCT with current enrollment at n=121 with complete 1-year follow-up. The primary outcome was cost-effectiveness, expressed as the incremental cost-effectiveness ratio. Secondary outcomes were health-related utility, presented as quality-adjusted life-years (QALYs), and cost, calculated in US dollars. Analysis was conducted following Second Panel on Cost-Effectiveness Health and Medicine recommendations. The base case analysis utilized SF-36 survey data from the RCT. Both cost and QALY outcomes were discounted at a yearly rate of 3% to reflect their present value. A cohort Markov model was constructed to analyze perioperative and postoperative costs and QALYs for both TOPS™ and control groups. Scenario, probabilistic, and threshold sensitivity analyses were conducted to determine model discrimination and calibration.

**Results:** The primary time horizon used to estimate cost and health utility was 2 years after index surgery. From a health system perspective, assuming a 50/50 split between Medicare and private payers, the TOPS™ cohort is cost-effective 2 years postoperatively (6158/QALY)comparedwithcontrol.At6yearsandbeyond,TOPS™becomesdominant,irrespectiveofpayermixandsurgicalsetting.Atwillingness−to−paythresholdsof100 000/QALY, 63% of all 5000 input parameter simulations favor TOPS, even with a $4000 upcharge vs TLIF.

**Discussion:** The novel TOPS™ device is cost-effective compared with TLIF and becomes the dominant economic strategy over time.

**Conclusions:** In the emerging, rapidly expanding field of value-based medicine, there will be an increased demand for these analyses, ensuring surgeons are empowered to make the best, most sustainable solutions for their patients and society.

## INTRODUCTION

Spinal fusion surgery is considered the gold standard for patients with moderate to severe lumbar spinal stenosis with degenerative spondylolisthesis (DS) that is not amenable to decompression alone.^1,2^ Well-constructed trials have produced data in support of fusion over laminectomy with notable improvement in SF-36 physical health-related quality of life (QOL),^1^ while others have concluded that decompression alone is noninferior to fusion.^3^ The debate becomes more nuanced when the Spine Patient Outcomes Research Trial (SPORT) group published their cost-effectiveness results concluding that fusion for DS was only moderately cost-effective compared with nonoperative conservative care.^4^ To further confound clinicians, others have looked at the magnitude of differences in the statistical inferences and whether these differences represent meaningful clinical changes, investigating topics such as minimum clinically important difference.^5,6^ Despite the often contradictory literature, clinical experience continues to play an important role in surgical decision-making. Many clinicians would opt to treat their patients with a decompression and fusion if they present with symptomatic lumbar stenosis and DS. Yet, in the era of motion preservation, many patients are concerned with adjacent segment disease and inquiring about alternatives to fusion, especially since people are in general living healthier, longer, and more active lifestyles. Motion-preserving alternatives that allow for concomitant decompression in DS therefore warrant serious consideration.

With increasing demand and declining payer reimbursement, societal tolerances for new technology requires large, often industry-sponsored randomized controlled trials (RCT) to assess efficacy, along with formal cost-utility analyses that comprehensively assess costs and QOL from a multitude of perspectives.^7–9^ Areas of interest related to QOL and motion preservation include reduced pain (and therefore narcotic use), improved patient-reported outcomes, reduced adjacent segment disease, faster return-to-work, and reduced reoperation rates.^10^ Under the current investigational device exemption (IDE) trial, the TOPS™ System (referred to as TOPS™, TOPS™ System, or Total Posterior Spine System) is indicated for stabilization, but not fusion, of 1 affected vertebral level between L2 and L5, following decompression surgery to alleviate leg pain with or without back pain stemming from all 3 of the following: (1) DS or retrolisthesis up to Grade 1; (2) moderate to severe lumbar spinal stenosis; and (3) thickening of the ligamentum flavum or scarring facet joint capsule. This study is intended to assess the cost-effectiveness of early data from the TOPS™ IDE.

Two approaches are commonly used to assess cost-effectiveness in healthcare: a simple incremental calculation or decision analytical modeling.^11^ A major drawback of this cost-accounting approach is its inability to describe relationships between clinical events, which impedes the prediction of how parameters change relative to one another. The TOPS™ System is essentially a lumbar facet arthroplasty motion-preserving device that is being compared to decompression and fusion. We therefore intend to perform a cost-utility analysis via decision analytical modeling using a Markov method to evaluate the cost-effectiveness of TOPS™ compared with the IDE control group, transforaminal lumbar interbody fusion (TLIF).

## METHODS

### Model Design

Patient informed consent and Institutional Review Board authorization were not required for this study since the patient cohort was extracted from an ongoing, multicenter RCT.^12^ Included patients were randomized preoperatively to receive either the experimental TOPS™ System or TLIF as the control arm. Initial enrollment goal is 240 with a 2:1 randomization favoring the experimental cohort. At the time of this analysis, mean age at presentation was 64 years (SD, ±8.26), and the total number of subjects enrolled was n=127 with at least 1 year of follow-up. The conventional time horizon used to estimate cost and health utility is 2 years. Additional postoperative periods examined in this analysis included 90 days, 1 year, 6 years, and 10 years.

The analysis was conducted to assess 2 principal outcome measures: cost and utility, in accordance with the Second Panel on Cost-Effectiveness Health and Medicine convened by the US Public Health Service.^13^ We adopted 2 commonly employed perspectives—societal and health system—as our base case cost assessment. The health system perspective accounts for direct medical costs alone, whereas the societal perspective accounts for both direct and indirect costs. Indirect costs are often referred to as productivity loss. Direct medical costs included operating room time, hospital stay, postoperative medications, follow-up visits (scheduled and unscheduled), surgery-related complications, device-related complications, and subsequent surgeries following such complications. Productivity loss was defined as lost workdays. Productivity loss was not computed for retired patients. All cost items were adjusted for inflation to 2020 dollars per the US medical care Consumer Price Index.^14,15^

Health-related utility outcome was expressed in quality-adjusted life-years (QALYs). The base case analysis utilized SF-12 data from the RCT, transforming this into weighted utilities based on SF-6D scores. Both cost and QALY outcomes were discounted at a yearly rate of 3% to reflect their present value.^15^ The cost-effectiveness outcome measure was calculated as the incremental cost-effectiveness ratio (ICER) for TOPS™ compared with TLIF. An ICER is the difference in cost divided by the difference in QALY for 2 interventions. A value under the commonly accepted US-based willingness-to-pay (WTP) threshold of $100 000 per QALY was considered cost-effective for TOPS™ compared with TLIF. Secondary thresholds, such as $50 000 per QALY and $150 000 per QALY, were also examined. Net monetary benefit was also calculated, representing the value of an intervention in monetary terms when a WTP threshold for a unit of benefit is applied.

### Health States

We constructed 5 health states to capture the level of pain and disability (and/or improvement) associated with the TOPS™ vs TLIF cohorts. They were constructed based on 2 dimensions for pain and disability: visual analog scale (VAS) and Oswestry disability index (ODI). In previous models, we found the correlation between VAS and ODI sufficient to anchor the SF-6D–constructed health states. By combining these dimensions using statistical regression, a clearer, more granular depiction of functional status can be obtained. The Kendall τ rank correlation between ODI and VAS was 0.655 (*P*<2.2^e-16^).

The health states created are depicted in **Supplementary Figure S1** and are divided by sections on the graph. Each section was determined from the regression equation and represents a different health state. Individual dots represent the observational units currently available in the data as of April 27, 2021.

### Markov Model

**Figure 1.**
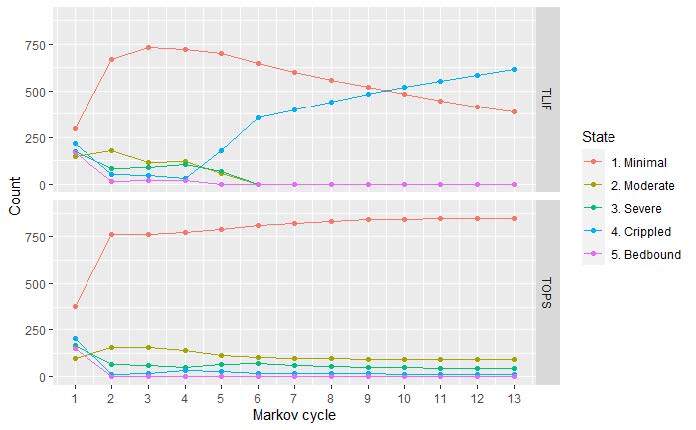
Markov Model Patient Cycles

A cohort Markov model (**Supplementary Figure S2**) was constructed to analyze perioperative and postoperative costs and health-related utility values for both TOPS™ and TLIF strategies. Five mutually exclusive Markov states depicting a patient’s health and work status are determined for each follow-up period. Each health state is associated with different costs and utility scores. Patients are redistributed across the 5 Markov states in each Markov cycle, attempting to parallel the postoperative course on a population level. The process of redistribution is controlled by 2 factors: (1) the preoperative distribution of health states; and (2) the transition probabilities between the health states (Tables 1 and 2). To better capture typical postoperative recovery trajectories, the model is designed with different cycle lengths, beginning with 1.5-month cycles and increasing to 3-month and eventually 12-month cycles (Figure 1).

**Table 1.** Input Parameters

**Parameter**	**Period**	**Value**		**Source**
**1. Health State (Level of Disability) at Index**				
Minimal	Initial State	0%		RCT (SF-12)
Moderate	Initial State	4%		
Severe	Initial State	27%		
Crippled	Initial State	39%		
Bedbound	Initial State	30%		
**2. Distribution of AE**				
		**TLIF**	**TOPS™**	RCT (AE and SE)
Minimal	Serious	1.12%	2.00%	
	Nonserious	7.87%	4.00%	
Moderate	Serious	0.00%	2.35%	
	Nonserious	2.86%	3.53%	
Severe	Serious	13.04%	2.56%	
	Nonserious	4.35%	20.51%	
Crippled	Serious	11.11%	26.67%	
	Nonserious	16.67%	33.33%	
Bedbound	Serious	25.00%	0.00%	
	Nonserious	25.00%	100.00%	
**3. Distribution of Subsequent Action Following AE Type**				
		**TLIF**	**TOPS™**	RCT (AE and SE)
Minimal	Serious—Supplemental Procedures	100.00%	77.78%	
	Serious—Surgery	0.00%	22.22%	
	Nonserious—Supplemental Procedures	92.86%	94.44%	
	Nonserious—Surgery	7.14%	5.56%	
Moderate	Serious—Supplemental Procedures	0.00%	0.50%	
	Serious—Surgery	0.00%	0.50%	
	Nonserious—Supplemental Procedures	100.00%	100.00%	
	Nonserious—Surgery	0.00%	0.00%	
Severe	Serious—Supplemental Procedures	66.67%	100.00%	
	Serious—Surgery	33.33%	0.00%	
	Nonserious—Supplemental Procedures	100.00%	100.00%	
	Nonserious—Surgery	0.00%	0.00%	
Crippled	Serious—Supplemental Procedures	50.00%	62.5%	
	Serious—Surgery	50.00%	37.5%	
	Nonserious—Supplemental Procedures	100.00%	100.00%	
	Nonserious—Surgery	0.00%	0.00%	
Bedbound	Serious—Supplemental Procedures	100.00%	0.00%	
	Serious—Surgery	0.00%	0.00%	
	Nonserious—Supplemental Procedures	100.00%	100.00%	
	Nonserious—Surgery	0.00%	0.00%	

Abbreviations: AE, adverse effects; RCT, randomized controlled trial; SE, side effects.

**Table 2.** Direct Costs

	**DRG/CPT**	**Medicare**
Ancillary procedure		
MRI lumbar spine w/o contrast	72148	$224.52
MRI lumbar spine w/wo contrast	72158	$378.41
CT lumbar spine w/ contrast	72132	$231.01
CT lumbar spine w/o contrast	72131	$182.36
CT lumbar spine w/wo contrast	72133	$272.46
Epidural steroid injection	62322	$88.66
Physical therapy	97110	$31.35
Office visit	99213	$51.90
Index operation		
Facility fee (TLIF)	DRG 460	$24 459
Facility fee (TOPS™)	DRG460+TOPS	$28 459
Laminectomy	63047	$1152
Posterior instrumentation	22840	$800
Insertion of biomechanical device	22853	$272
Allograft	20931	117
Fluoroscopy	77003	$100
Revision, fixation, or reoperation		
Facility fee (TLIF or TOPS™)	DRG 459	$40 822
Removal of device	22850	$758
Posterior instrumentation	22840	$800
Insertion of biomechanical device	22853	$272
Allograft	20931	117
Fluoroscopy	77003	$100

Abbreviation: DRG/CPT, Diagnosis-Related Group/Ambulatory Payment Classification; TLIF, transformational lumbar interbody fusion; w/wo, with or without.

Model input parameters are presented in Table 1. We considered 2 postoperative clinically pertinent events: (1) adverse events (AEs) (serious and nonserious) requiring nonoperative procedures/interventions and (2) AEs (serious and nonserious) requiring reoperation. All AEs were stratified by health state and RCT cohort. All costs associated with AEs were calculated accordingly (Table 1).

### Transition Probability Parameters

**Direct costs:** Direct costs were taken from Medicare and private data sources (Table 2). Total costs were compared with recent published health economic TLIF literature to verify accuracy. The base case model utilizes a 50/50 split of Medicare and private rates to better reflect a more realistic patient population. This split is consistent with the age demographics found in the TOPS™ IDE study. Supplemental procedures (eg, office visits, physical therapy, imaging, injection) were also considered in the model. For the TOPS™ device cost, we assumed a $4000 upcharge over TLIF. Pertinent medication use was extracted from the RCT data and coded. We aggregated costs per health state for each time point to determine total costs for each disability level. Average wholesale price was found for each medication using Redbook MarketScan.^16^ Table 2 highlights direct cost inputs; **Supplementary Table S4** illustrates modeling results for average medication costs by time period, by health state, and per trial participant.

**Indirect costs:** Work status from the Zurich Claudication Questionnaire (CQ16) was used to capture indirect costs. Work status is categorized into either “work with no restriction,” “unable to work,” and “not working for unrelated reasons” (ie, unemployed, student, or retired). We applied 2020 US national average annual wages to calculate productivity loss associated with health state transitions over time. Productivity loss is included in the scenarios analyzed from a societal perspective (**Supplementary Table S3**).

### Base Case Scenario and Sensitivity Analysis

The base case results are presented for a specific scenario in which a set of assumptions are employed. The assumptions for the base case scenario include: (1) costs and health benefits are accrued within a 2-year analytical time horizon; (2) insurers represent 50% Medicare and 50% private for our analyzed sample; (3) index surgery for a patient receiving TOPS™ is $4000 greater than TLIF; and (4) all initial surgeries were completed in hospital inpatient settings. These assumptions have substantial effects on the cost-effectiveness outcome. We therefore conducted a scenario sensitivity analysis, varying payer mix and setting.

Inherent uncertainty is always associated with the input parameters used in a base case cost-effectiveness analysis. Therefore, a one-way sensitivity analysis (OWSA) was also used to identify the parameters associated with the greatest uncertainty and influence on our conclusions (**Supplementary Figures S3, S4, and S5**). In the OWSA, we varied each of the 38 input parameters individually. Each parameter is varied by ±20% of its base case value.

**Figure 2.**
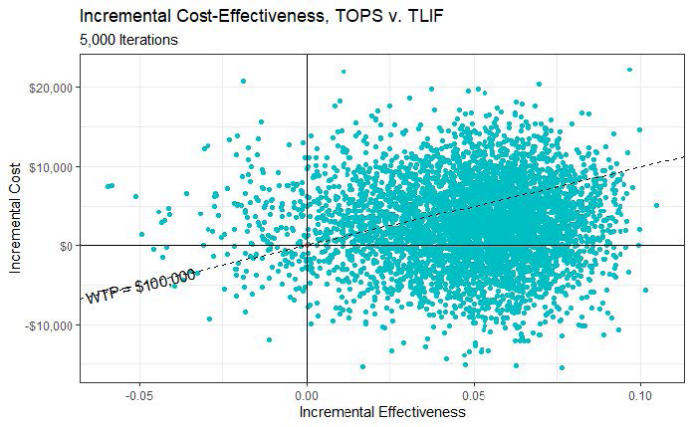
Cost-effectiveness Scatterplot^a^ Abbreviations: CE, cost-effectiveness; TLIF, transformational lumbar interbody fusion. ^a^ The y-axis indicates the percentage of the 5000 iterations in which the strategy is considered cost-effective given a specific WTP threshold. For example, the probability for TOPS at the WTP of $100 000 per QALY gain is approximately 63.1%, meaning that TOPS is the more cost-effective strategy in ~3155 (5000*63.1%) iterations.

A probabilistic sensitivity analysis (PSA) was the final model calibration (Figure 2). All input parameters (the 34 costs and 5 utilities individually tested in OWSA) are varied simultaneously to assess cost-effectiveness outcomes in response to collective parameter uncertainty. This uncertainty was characterized by probability distributions.^17^ Beta distributions (best fit for binomial data) were assigned to all probability parameters based on their point estimates and 95% confidence intervals derived from the trial data. Gamma distributions were used for cost items with SD, by convention being 15.3% (30%/1.96). Gamma distributions were also assigned to decrements in QALYs, with lower and upper bounds of 0 and 1, respectively. A Monte Carlo simulation with 5000 iterations was then run to determine likelihood of cost-effectiveness.

## RESULTS

### Base Case

Most patients (93%) began with high ODI and VAS scores, falling within the 3 worst health states (Table 1). At this early phase in the analysis, AEs were rare for both groups, although a nonsignificant advantage in AEs was observed for TOPS™ vs TLIF. From the health systems perspective, at 2 years, TOPS™ incurred a $300 greater cost than TLIF while imparting 0.0489 additional QALYs (Table 3). The 2-year ICER for TOPS™ vs TLIF was therefore 6157 $/QALY, significantly lower than the most conservative US willingness-to-pay (WTP) threshold of $50 000/QALY. Notably, at 1 year, the ICER is less favorable at $61 445/QALY, but this value continues to remain below the more current and acceptable WTP threshold of $100 000/QALY. When the data are extrapolated 6 years and beyond, the ICER becomes negative, suggesting economic dominance. From a societal perspective, TOPS™ reduced costs by $1832 over 2 years with the same QALY gain of 0.489. This negative ICER again alludes to an economically dominant strategy (Table 3). At 1 year, the ICER remains cost-effective at $25 376/QALY. Net monetary benefit (NMB) demonstrates a cost savings for TOPS™ over TLIF at the WTP of $50 000/QALY at 2 years -$2142 and $4275 for the health system and societal perspectives, respectively (**Supplementary Table S5**). Small differences in work status between groups were observed. Modeled at 2 years, per health state, a trend favoring the TOPS™ group was appreciated, especially in the “unable to work” group (**Supplementary Table S3**).

**Table 3.** Base Case Results with Medicare and Private Rates, Both Perspectives

**TOPS™**	**Control**						
**Time Horizon**	**Cost^a^**	**QALY**	**Cost**	**QALY**	**∆Cost^b^**	**∆QALY^c^**	**ICER,^d^ $ per QALY**
**Health systems**							
90-day	$41 513	0.1759	$40 032	0.1697	$1481	0.0063	$236 407
1-year	$43 445	0.7171	$42 409	0.7003	$1036	0.0169	$61 446
2-year (base case)	$44 763	1.4142	$44 462	1.3653	$300	0.0489	$6158
6-year (extrapolated)	$49 349	4.0382	$56 076	3.6848	-$6727	0.3534	Dominant
10-year (extrapolated)	$53 320	6.3865	$68 867	5.6009	-$15 546	0.7856	Dominant
**Societal**							
90-day	$41 769	0.1759	$40 419	0.1697	$1349	0.0063	$215 357
1-year	$45 170	0.7171	$44 742	0.7003	$427	0.0169	$25 377
2-year (base case)	$48 330	1.4142	$50 162	1.3653	-$1832	0.0489	Dominant
6-year (extrapolated)	$58 608	4.0382	$81 771	3.6848	-$23 162	0.3534	Dominant
10-year (extrapolated)	$67 075	6.3865	$118 371	5.6009	-$51 296	0.7856	Dominant

^a^ Includes TOPS™ cost in the initial surgery.

^b^ ∆Cost = TOPS™ Cost - Control Cost.

^c^ ∆QALY = TOPS™ QALY - Control QALY.

^d^ ICER = ∆Cost / ∆QALY; “Dominant” indicates that TOPS™ costs less while yielding a higher QALY.

### Sensitivity Analysis

When we allocated 50% of the initial surgeries to hospital inpatient and 50% to hospital outpatient (**Supplementary Table S6, Panel 6**), the ICER for TOPS™ at 2 years is greater than the original base case ($87 607/QALY vs $6158/QALY). Similarly, if all initial surgeries were conducted in an outpatient setting instead (**Supplementary Table S6, Panel 5**), the TOPS™ strategy (ICER, $169 057/QALY; NMB at $100 000 WTP, -$3375) is worse compared with the base case. If all patients were assumed to be Medicare enrollees (**Supplementary Table S6, Panel 3**), then the ICER ($61 046/QALY) and NMB at $100 000 WTP ($1904) is again worse than the base case of 50/50 Medicare/privately insured (ICER, $6158/QALY; NMB, $4586). However, a patient population considering only private payer rates (**Supplementary Table S6, Panel 4**) fares better than the base case analysis (ICER, -$48 730/QALY; NMB, $7268).

In the OWSA (**Supplementary Figures S3, S4, and 5**), our model was reliably stable even when the input parameters were varied by ±20%. The cost difference between strategies is centered around $300 (Table 3, base case ∆cost) and is largely positive despite changes to facility fees. The effect difference is also positively stable.

**Figure 3.**
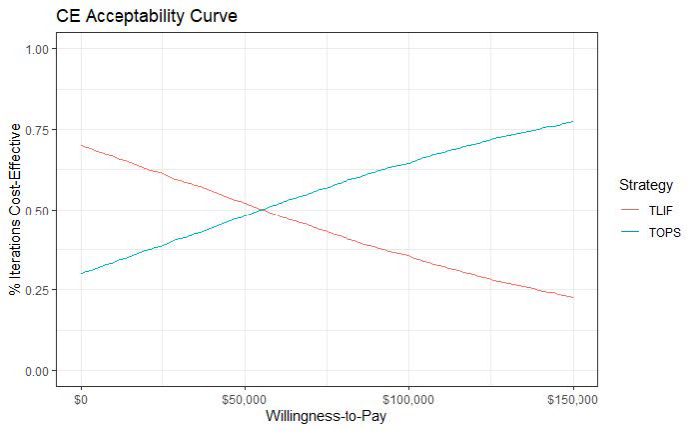
Cost-effectiveness Acceptability Curves^a^ Abbreviations: CE, cost-effectiveness; TLIF, transformational lumbar interbody fusion. ^a^ The y-axis indicates the percentage of the 5000 iterations in which the strategy is considered cost-effective given a specific WTP threshold. For example, the probability for TOPS at the WTP of $100 000 per QALY gain is approximately 63.1%, meaning that TOPS is the more cost-effective strategy in ~3155 (5000*63.1%) iterations.

In the PSA, cost differences between TOPS™ and TLIF range from ~-$20 000 up to ~$20 000, and QALY difference ranges from ~-0.06 to ~0.11 (Figure 2). Each point represents the result of 1 simulation out of 5000. Points below the WTP line of $100 000 per QALY gained indicate that the TOPS™ strategy is cost-effective over the control. As depicted in the figure, more than half of the 5000 iterations have the TOPS™ strategy as being cost-effective over the control (the majority of points lie under the dotted line). The cost-effectiveness acceptability curve illustrates the PSA results differently (Figure 3). At a WTP equal to $100 000, approximately 63.1% of the simulations have TOPS™ as cost-effective over TLIF.

## DISCUSSION

At $6157/QALY, the 2-year ICER for TOPS™ vs TLIF is considered highly cost-effective. This ICER compares to cataract surgery, often considered to be one of the most cost-effective procedures globally.^18^ This is also similar to the cost-effectiveness of epilepsy surgery, $4000-$20 000/QALY; hip arthroplasty, $2300-$4800/QALY; knee arthroplasty, $6500-$12 700/QALY; and defibrillator implantation, $700-$23 000/QALY.^18,19^ This is compelling since the TOPS™ strategy also becomes dominant at 2 years from the societal perspective. Economic dominance is interpreted as increased quality/utility for less cost, which is compelling when assessing the sustainability of novel interventions. Unfortunately, payers and policy makers often overlook indirect costs. We contend that this has long been an unacceptable and inappropriate convention. Third-party payer policies are not always congruent with best practice recommendations or financially sustainable solutions.

In this preliminary analysis, there was a trend toward fewer AEs in the TOPS™ group compared with TLIF. When examining elective surgery for degenerative lumbar spine disease, Chotai et al^20^ found that patient with 90-day complications had significantly higher hospital cost ($20 328 vs $15 388, *P*<.0001). Family members also took days off to care for the patient, or a caregiver was hired, further increasing indirect costs. Decreased QALYs gained was also observed, resulting in ICERs of $70 822/QALY and $45 831/QALY, for patients with and without 90-day complications, respectively.^20^ Interestingly, Tso et al^21^ reported a projected lifetime incremental cost-utility ratio of $2307/QALY gained for lumbar decompression and $7153/QALY gained for decompression with fusion. This difference may be due to increased fusion costs but also the declining efficacy and QALYs gained, also seen in our analysis, that is typical of this cohort over time.

It is critical that this analysis be considered in the context of its limitations. As with other complex statistical approaches, the Markov model is conditional on the present state alone; future and past events are independent. With disease processes, it is rarely plausible to assume that a patient’s transition to another health state was entirely independent of a previous health state. The model also assumed that surgical cohorts began in similar health states, which is likely acceptable because of the trial randomization and nonsignificant differences in baseline characteristics. We also recognize that some cost data were not ascertainable. As it is problematic to use hospital charge data to conduct a cost-effective analysis, we used Medicare and Humana Diagnosis-Related Group/Ambulatory Payment Classification rates as representations of public and private payers. As a result, differences in parameters (such as operating room time and length of stay) were not used. The authors recognize this as a limitation; however, it is noteworthy that differences in these parameters were not statistically different in the clinical manuscript and would therefore not impact our cost calculations. Medication-related costs were estimated from the average wholesale price. Although this estimate is considered appropriate, it is impossible to determine if it overestimated or underestimated costs for both groups. Productivity loss was also a significant contributor to cost, but this analysis was unable to include factors such as transportation costs, caregiver time/responsibilities, and educational days missed as these were not captured in the primary dataset/trial.

To address modeling limitations and inherent uncertainty, our analysis was rigorously tested. In the scenario sensitivity analysis, assuming a WTP of $100 000, cost-effectiveness was achieved in 3 of 8 scenarios by 1 year and in 5 of 8 scenarios by 2 years. Utilities are not reliably affected by changes in insurance or surgical setting (the latter remains debatable) and are favorable for TOPS™ in every scenario and timepoint. In the OWSA, the combined cost and utility inputs rank together. This reaffirms our base case results that at 2 years, differences in the 2 strategies are going to be positive for both costs and utilities. The model is susceptible to some variability, however, and overall acceptability of the intervention will invariably depend significantly on the WTP threshold. The results of the PSA were equally compelling. The ICER for TOPS™ consistently fell below current pricing and societal tolerances for novel healthcare technologies.

Collectively, TOPS™ appears to be a highly cost-effective surgical modality compared with TLIF as a motion-preserving, non-fusion alternative for the treatment of grade 1 DS with lumbar stenosis. In the United States, health care expenditure in 2019 was calculated at $3.8 trillion dollars, or 17.7% of the gross domestic product.^22^ This is estimated to increase to $6.2 trillion dollars or 19.7% of the US gross domestic product by 2028.^22^ The reality of increasing healthcare costs despite limited resources now pervades society. Cost-effective analyses are no longer novel but rather essential to critically evaluating novel technologies. This is the first instance of a lumbar facet arthroplasty device demonstrating cost-effectiveness over fusion. Since the TOPS™ System yields greater QOL at a lower total cost over time, it deserves serious attention. Undeniably, this cost-effectiveness analysis is preliminary and the first comprehensive analysis of the IDE RCT data. We plan to update the model as longer-term safety and efficacy data continue to be collected with formal recommendations regarding US market adoption at that time.

### Disclosures

The analysis of IDE trial data to conduct a cost-effectiveness evaluation of the TOPS™ System was funded by Premia Spine Inc. Premia Spine directly paid Neuronomics LLC, a healthcare economics think tank. JDA is the President/CEO of Neuronomics. Premia Spine had no input regarding the creation, critical revision, or fundamental production of this manuscript.

### Author Contributions

JDA carried out conception and design, analysis and interpretation of data, drafting of the manuscript, critical revision of the manuscript, statistical analysis, obtaining funding, administrative support, and supervision. AV carried out analysis and interpretation of data, drafting of the manuscript, critical revision of the manuscript, statistical analysis, administrative support, and supervision. YB carried out analysis and interpretation of data, critical revision of the manuscript, administrative support, and supervision. TL carried out analysis and interpretation of data, critical revision of the manuscript, administrative support, and supervision. KK carried out analysis and interpretation of data, critical revision of the manuscript, administrative support, and supervision. JPJ carried out analysis and interpretation of data, drafting of manuscript, critical revision of the manuscript, administrative support, and supervision.

## Supplementary Material

Supplementary Online Material

Supplementary Online Material
