# Supplementary material for: A Prospective Study of Lumbar Facet Arthroplasty in the Treatment of Degenerative Spondylolisthesis and Stenosis: Early Cost-effective Assessment from the Total Posterior Spine System (TOPS™) IDE Study
Source: J Health Econ Outcomes Res. 2022 Mar 25;9(1):82–9. doi: 10.36469/001c.33035 (PMC9132256; doi:10.36469/001c.33035)
Supplement: Supplementary Online Material [file jheor_2022_9_1_33035_86119.pdf]

Journal of Health Economics and Outcomes Research

Online Supplementary Material

Ament JD, Vokshoor A, Badr Y, et al. A prospective study of lumbar facet arthroplasty in the treatment of degenerative spondylolisthesis and stenosis: early cost-effective assessment from the Total Posterior Spine System (TOPS™) IDE Study. *JHEOR*. **2022;9(1):82-89.** doi:10.36469/jheor.2022.33035

Figure S1: Mapping of Health States

Figure S2: Markov Schematic

Figure S3: One-way Sensitivity Analysis Results: Costs of TOPS™ vs Control

Figure S4: One-way Sensitivity Analysis Results: Effect of TOPS™ vs Control

Figure S5: One-way Sensitivity Analysis Results: Incremental Cost-effectiveness Ratio of TOPS™ vs Control

Table S1: TOPS™ Transition Probabilities

Table S2: TLIF Transition Probabilities

Table S3: Productivity Loss Category Probabilities by Time Point

Table S4: Average Medication Costs by Health State per Individual

Table S5: Net Monetary Benefit, Both Perspectives

Table S6: Cost-Effectiveness of TOPS™ vs TLIF in Alternative Scenarios

This supplementary material has been provided by the authors to give readers additional information about their work.

This is an open-access article distributed under the terms of the Creative Commons Attribution 4.0 International License (CCBY-4.0). View this license's legal deed at http://creativecommons.org/licenses/by/4.0 and legal code at http://creativecommons.org/licenses/by/4.0/legalcode for more information.

Figure S1. Mapping of Health States

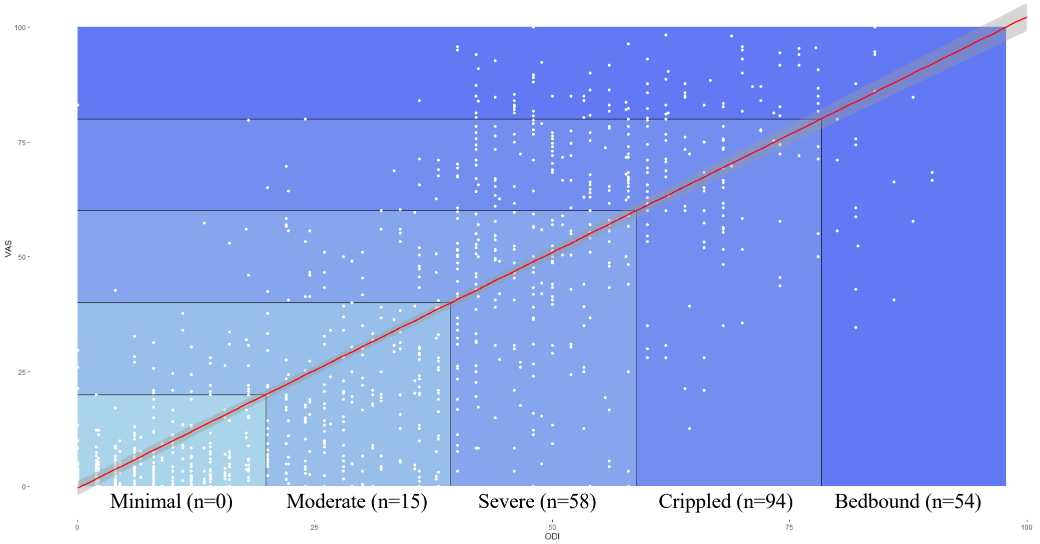

Figure S2. Markov Schematic

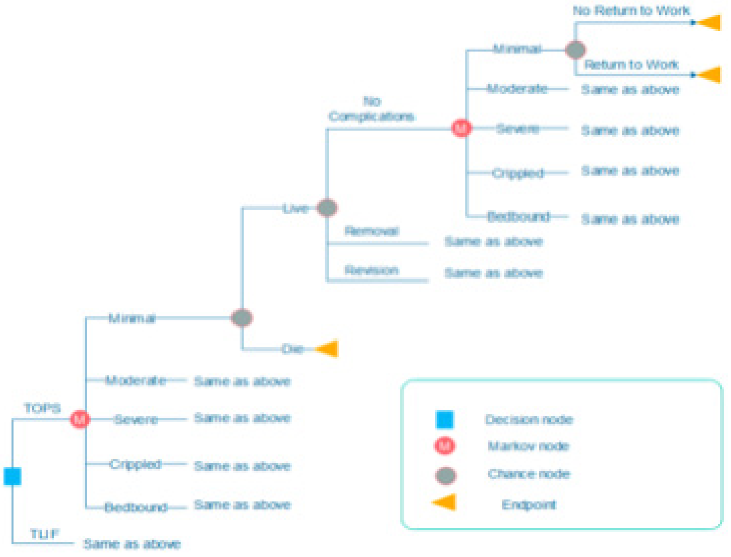

Abbreviation: TLIP, transformational lumbar interbody fusion.

Figure S3. One-way Sensitivity Analysis Results: Costs of TOPS™ vs Control

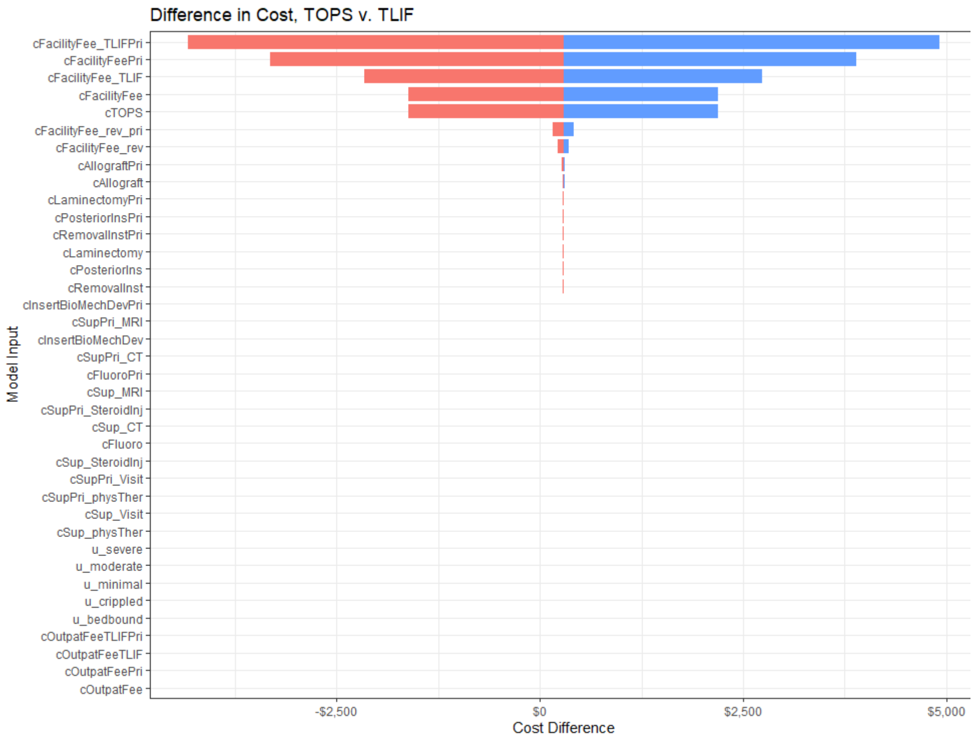

Abbreviation: TLIP, transformational lumbar interbody fusion.

Figure S4. One-way Sensitivity Analysis Results: Effect of TOPS™ vs Control

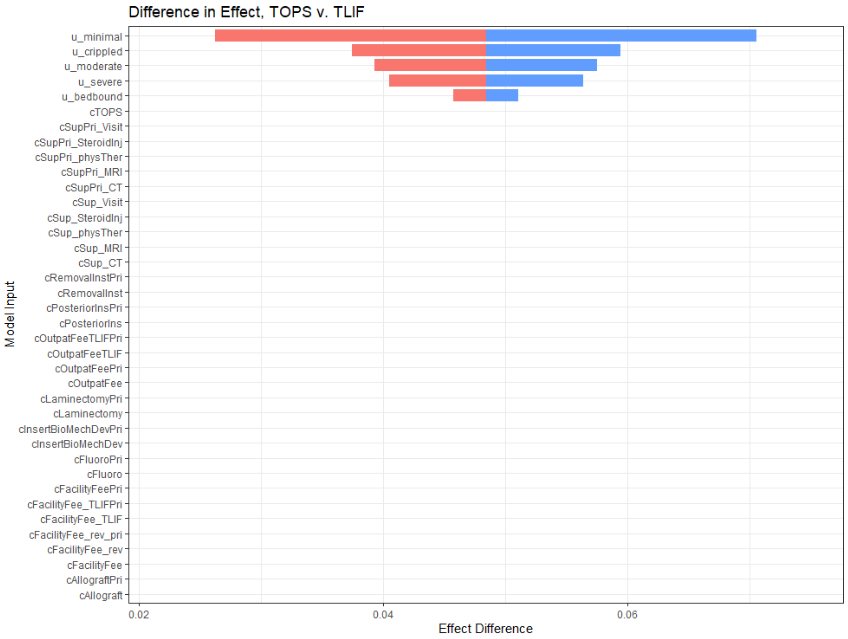

Abbreviation: TLIP, transformational lumbar interbody fusion.

Figure S5. One-way Sensitivity Analysis Results: Effect of TOPS™ vs Control

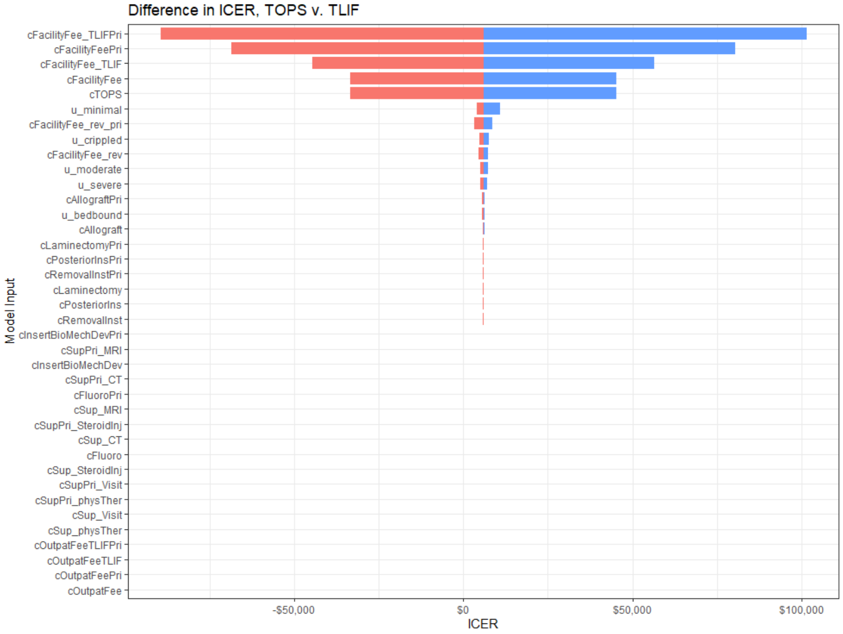

Abbreviation: TLIP, transformational lumbar interbody fusion.

Table S1. TOPS™ Transition Probabilities

**From Health State**	**Transit to Health State**					**Time Range**
	**Minimal**	**Moderate**	**Severe**	**Crippled**	**Bedbound**	
Minimal	1.000	0.000	0.000	0.000	0.000	Surgery-6W
	0.889	0.081	0.030	0.000	0.000	6W-3M
	0.891	0.076	0.033	0.000	0.000	3M-6M
	0.929	0.043	0.014	0.014	0.000	6M-12M
	0.947	0.053	0.000	0.000	0.000	12M-24M+
Moderate	1.000	0.000	0.000	0.000	0.000	Surgery-6W
	0.522	0.348	0.130	0.000	0.000	6W-3M
	0.500	0.350	0.100	0.050	0.000	3M-6M
	0.444	0.278	0.167	0.111	0.000	6M-12M
	0.333	0.333	0.167	0.167	0.000	12M-24M+
Severe	0.775	0.225	0.000	0.000	0.000	Surgery-6W
	0.000	0.625	0.250	0.125	0.000	6W-3M
	0.125	0.500	0.125	0.250	0.000	3M-6M
	0.000	1.000	0.000	0.000	0.000	6M-12M
	0.333	0.333	0.333	0.000	0.000	12M-24M+
Crippled	0.729	0.186	0.085	0.000	0.000	Surgery-6W
	0.000	0.000	1.000	0.000	0.000	6W-3M
	0.000	1.000	0.000	0.000	0.000	3M-6M
	0.000	0.000	0.500	0.500	0.000	6M-12M
	0.000	0.000	1.000	0.000	0.000	12M-24M+
Bedbound	0.758	0.091	0.091	0.061	0.000	Surgery-6W
	0.000	0.000	0.000	0.000	1.000	6W-3M
	0.000	0.000	0.000	0.000	1.000	3M-6M
	0.000	0.000	0.000	0.000	1.000	6M-12M
	0.000	0.000	0.000	0.000	1.000	12M-24M+

Abbreviations: M, months; W, weeks.

Table S2. TLIF Transition Probabilities

From Health State	Transit to Health State					Time Range
	**Minimal**	**Moderate**	**Severe**	**Crippled**	**Bedbound**	
Minimal	1.000	0.000	0.000	0.000	0.000	Surgery-6W
	0.919	0.054	0.027	0.000	0.000	6W-3M
	0.868	0.026	0.079	0.000	0.026	3M-6M
	0.846	0.115	0.038	0.000	0.000	6M-12M
	0.929	0.000	0.000	0.071	0.000	12M-24M+
Moderate	0.714	0.143	0.143	0.000	0.000	Surgery-6W
	0.692	0.231	0.077	0.000	0.000	6W-3M
	0.600	0.200	0.200	0.000	0.000	3M-6M
	0.250	0.250	0.500	0.000	0.000	6M-12M
	0.000	0.000	0.000	1.000	0.000	12M-24M+
Severe	0.647	0.294	0.059	0.000	0.000	Surgery-6W
	0.000	0.000	0.800	0.200	0.000	6W-3M
	0.200	0.600	0.000	0.000	0.200	3M-6M
	0.500	0.000	0.500	0.000	0.000	6M-12M
	0.000	0.000	0.000	1.000	0.000	12M-24M+
Crippled	0.643	0.179	0.036	0.107	0.036	Surgery-6W
	0.000	0.333	0.000	0.667	0.000	6W-3M
	0.000	0.500	0.000	0.500	0.000	3M-6M
	0.000	0.000	0.000	1.000	0.000	6M-12M
	0.000	0.000	0.000	1.000	0.000	12M-24M+
Bedbound	0.467	0.333	0.133	0.000	0.067	Surgery-6W
	0.500	0.000	0.000	0.500	0.000	6W-3M
	0.000	0.000	0.000	0.000	1.000	3M-6M
	1.000	0.000	0.000	0.000	0.000	6M-12M
	0.000	0.000	0.000	0.000	1.000	12M-24M+

Abbreviations: M, months; TLIF, transformational lumbar interbody fusion; W, weeks.

Table S3. Productivity Loss Category Probabilities by Time Point

Parameter	Period	Value	
1. Work without restriction		**TLIF**	**TOPS™**
Minimal	All time	93.94%	95.74%
Moderate	All time	60.00%	60.78%
Severe	All time	23.81%	23.40%
Crippled	All time	10.00%	8.89%
Bedbound	All time	6.25%	0.00%
2. Unable to work		**TLIF**	**TOPS™**
Minimal	All time	6.06%	3.88%
Moderate	All time	30.00%	33.33%
Severe	All time	23.81%	17.02%
Crippled	All time	15.00%	2.22%
Bedbound	All time	6.25%	3.23%
3. Work N/A		**TLIF**	**TOPS™**
Minimal	All time	0.00%	0.39%
Moderate	All time	10.00%	5.88%
Severe	All time	52.38%	59.57%
Crippled	All time	75.00%	88.89%
Bedbound	All time	87.50%	96.77%

Abbreviation: TLIF, transformational lumbar interbody fusion.

Table S4. Average Medication Costs by Health State per Individual

Period/Parameter	Value		Source
Surgery-6W	**TLIF**	**TOPS™**	
Minimal			
Moderate			
Severe	$149	$121	
Crippled			
Bedbound			
6W+			RCT
Minimal	$0	$76	
Moderate	$55	$58	
Severe	$50	$72	
Crippled	$55	$134	
Bedbound	$0	$12	

Abbreviations: RCT, randomized controlled trial; TLIF, transformational lumbar interbody fusion; W, weeks.

Table S5. Net Monetary Benefit, Both Perspectives

		TOPS™		Control		NMB, Based on 3 WTP Thresholds[b]		
	Time Horizon	Cost [a]	QALY	Cost	QALY	WTP=$50 000	WTP=$100 000	WTP=$150 000
Health systems	90-day	$41 513.08	0.1759	$40 031.79	0.1697	-$1168.00	-$854.71	-$541.42
	1-year	$43 444.75	0.7171	$42 408.64	0.7003	-$193.00	$650.11	$1493.22
	2-year	$44 762.57	1.4142	$44 461.65	1.3653	$2142.46	$4585.85	$7029.23
	6-year	$49 348.65	4.0382	$56 075.82	3.6848	$24 395.90	$42 064.63	$59 733.37
	10-year	$53 320.47	6.3865	$68 867.20	5.6009	$54 826.89	$94 107.05	$133 387.21
Societal	90-day	$41 768.70	0.1759	$40 419.30	0.1697	-$1036.10	-$722.81	-$409.52
	1-year	$45 169.82	0.7171	$44 741.92	0.7003	$415.20	$1258.31	$2101.42
	2-year	$48 329.80	1.4142	$50 162.08	1.3653	$4275.66	$6719.05	$9162.43
	6-year	$58 608.36	4.0382	$81 771.23	3.6848	$40 831.60	$58 500.34	$76 169.07
	10-year	$67 074.61	6.3865	$118 371.24	5.6009	$90 576.79	$129 856.95	$169 137.11

Abbreviations: NMB, net monetary benefit; QALY, quality-adjusted life-year; WTP, willingness-to-pay.

[a] Includes TOPS™ cost in the initial surgery.

[b] NMB = ΔQALY *WTP threshold - ΔCost.

Table S6. Cost-effectiveness of TOPS™ vs TLIF in Alternative Scenarios

Scenario		TOPS™		TLIF		Difference		Results		Net Monetary Benefit		
	**Time point**	**Cost**	**Utilities**	**Cost**	**Utilities**	**Cost**	**Utilities**	**ICER**	**Status**	**$50,000**	**$100,000**	**$150,000**
1. Health systems 50/50 insurance inpatient (base case)	3-month	$41 513.08	0.1759	$40 031.79	0.1697	$1481	0.0063	$236 407	Undominated	-$1168.00	-$854.71	-$541.42
	1-year	$43 444.75	0.7171	$42 408.64	0.7003	$1036	0.0169	$61 446	Undominated	-$193.00	$650.11	$1493.22
	2-year	$44 762.57	1.4142	$44 461.65	1.3653	$301	0.0489	$6158	Undominated	$2142.46	$4585.85	$7029.23
	6-year	$49 348.65	4.0382	$56 075.82	3.6848	-$6727	0.3534	-$19 037	Dominant	$24 395.90	$42 064.63	$59 733.37
	10-year	$53 320.47	6.3865	$68 867.20	5.6009	-$15 547	0.7856	-$19 790	Dominant	$54 826.89	$94 107.05	$133 387.21
	**Time point**	**Cost**	**Utilities**	**Cost**	**Utilities**	**Cost**	**Utilities**	**ICER**	**Status**	**$50 000**	**$100 000**	**$150 000**
2. Societal 50/50 insurance inpatient (base case)	3-month	$41 768.70	0.1759	$40 419.30	0.1697	$1349	0.0063	$215 357	Undominated	-$1036.10	-$722.81	-$409.52
	1-year	$45 169.82	0.7171	$44 741.92	0.7003	$428	0.0169	$25 377	Undominated	$415.20	$1258.31	$2101.42
	2-year	$48 329.80	1.4142	$50 162.08	1.3653	-$1832	0.0489	-$37 495	Dominant	$4275.66	$6719.05	$9162.43
	6-year	$58 608.36	4.0382	$81 771.23	3.6848	-$23 163	0.3534	-$65 548	Dominant	$40 831.60	$58 500.34	$76 169.07
	10-year	$67 074.61	6.3865	$118 371.24	5.6009	-$51 297	0.7856	-$65 296	Dominant	$90 576.79	$129 856.95	$169 137.11
	**Time point**	**Cost**	**Utilities**	**Cost**	**Utilities**	**Cost**	**Utilities**	**ICER**	**Status**	**$50 000**	**$100 000**	**$150 000**
3. Health systems 100% Medicare inpatient	3-month	$31 721.58	0.1759	$27 782.30	0.1697	$3939	0.0063	$628 690	Undominated	-$3625.98	-$3312.69	-$2999.40
	1-year	$33 393.56	0.7171	$29 815.58	0.7003	$3578	0.0169	$212 190	Undominated	-$2734.87	-$1891.76	-$1048.66
	2-year	$34 579.61	1.4142	$31 596.46	1.3653	$2983	0.0489	$61 046	Undominated	-$539.77	$1903.62	$4347.00
	6-year	$38 731.64	4.0382	$41 417.04	3.6848	-$2685	0.3534	-$7599	Dominant	$20 354.13	$38 022.86	$55 691.59
	10-year	$42 332.92	6.3865	$52 146.70	5.6009	-$9814	0.7856	-$12 492	Dominant	$49 093.94	$88 374.10	$127 654.25
	**Time point**	**Cost**	**Utilities**	**Cost**	**Utilities**	**Cost**	**Utilities**	**ICER**	**Status**	**$50 000**	**$100 000**	**$150 000**
4. Health systems 100% private inpatient	3-month	$51 304.59	0.1759	$52 281.28	0.1697	-$977	0.0063	-$155 875	Dominant	$1289.98	$1603.28	$1916.57
	1-year	$53 495.93	0.7171	$55 001.69	0.7003	-$1506	0.0169	-$89 298	Dominant	$2348.87	$3191.98	$4035.09
	2-year	$54 945.53	1.4142	$57 326.85	1.3653	-$2381	0.0489	-$48 730	Dominant	$4824.70	$7268.08	$9711.47
	6-year	$59 965.67	4.0382	$70 734.61	3.6848	-$10 769	0.3534	-$30 475	Dominant	$28 437.67	$46 106.41	$63 775.14
	10-year	$64 308.01	6.3865	$85 587.70	5.6009	-$21 280	0.7856	-$27 087	Dominant	$60 559.85	$99 840.01	$139 120.17
	**Time point**	**Cost**	**Utilities**	**Cost**	**Utilities**	**Cost**	**Utilities**	**ICER**	**Status**	**$50 000**	**$100 000**	**$150 000**
5. Health systems 50/50 insurance outpatient	3-month	$32 553.08	0.1759	$23 111.29	0.1697	$9442	0.0063	$1 506 866	Undominated	-$9128.50	-$8815.21	-$8501.92
	1-year	$34 484.75	0.7171	$25 488.14	0.7003	$8997	0.0169	$533 538	Undominated	-$8153.50	-$7310.39	-$6467.28
	2-year	$35 802.57	1.4142	$27 541.15	1.3653	$8261	0.0489	$169 057	Undominated	-$5818.04	-$3374.65	-$931.27
	6-year	$40 388.65	4.0382	$39 155.32	3.6848	$1233	0.3534	$3490	Undominated	$16 435.40	$34 104.13	$51 772.87
	10-year	$44 360.47	6.3865	$51 946.70	5.6009	-$7586	0.7856	-$9657	Dominant	$46 866.39	$86 146.55	$125 426.71

	Time point	Cost	Utilities	Cost	Utilities	Cost	Utilities	ICER	Status	$50 000	$100 000	$150 000
6. Health systems 50/50 insurance 50/50	3-month	$37 033.08	0.1759	$31 571.54	0.1697	$5462	0.0063	$871 637	Undominated	-$5148.25	-$4834.96	-$4521.67
	1-year	$38 964.75	0.7171	$33 948.39	0.7003	$5016	0.0169	$297 492	Undominated	-$4173.25	-$3330.14	-$2487.03
	2-year	$40 282.57	1.4142	$36 001.40	1.3653	$4281	0.0489	$87 607	Undominated	-$1837.79	$605.60	$3048.98
	6-year	$44 868.65	4.0382	$47 615.57	3.6848	-$2747	0.3534	-$7773	Dominant	$20 415.65	$38 084.38	$55 753.12
	10-year	$48 840.47	6.3865	$60 406.95	5.6009	-$11 566	0.7856	-$14 723	Dominant	$50 846.64	$90 126.80	$129 406.96
	**Time point**	**Cost**	**Utilities**	**Cost**	**Utilities**	**Cost**	**Utilities**	**ICER**	**Status**	**$50 000**	**$100 000**	**$150 000**
7. Health systems 100% Medicare outpatient	3-month	$25 700.58	0.1759	$16 252.30	0.1697	$9448	0.0063	$1 507 901	Undominated	-$9134.98	-$8821.69	-$8508.40
	1-year	$27 372.56	0.7171	$18 285.58	0.7003	$9087	0.0169	$538 897	Undominated	-$8243.87	-$7400.76	-$6557.66
	2-year	$28 558.61	1.4142	$20 066.46	1.3653	$8492	0.0489	$173 778	Undominated	-$6048.77	-$3605.38	-$1162.00
	6-year	$32 710.64	4.0382	$29 887.04	3.6848	$2824	0.3534	$7990	Undominated	$14 845.13	$32 513.86	$50 182.59
	10-year	$36 311.92	6.3865	$40 616.70	5.6009	-$4305	0.7856	-$5480	Dominant	$43 584.94	$82 865.10	$122 145.25
	**Time point**	**Cost**	**Utilities**	**Cost**	**Utilities**	**Cost**	**Utilities**	**ICER**	**Status**	**$50 000**	**$100 000**	**$150 000**
8. Health systems 100% private outpatient	3-month	$39 405.59	0.1759	$29 970.28	0.1697	$9435	0.0063	$1 505 832	Undominated	-$9122.02	-$8808.72	-$8495.43
	1-year	$41 596.93	0.7171	$32 690.69	0.7003	$8906	0.0169	$528 178	Undominated	-$8063.13	-$7220.02	-$6376.91
	2-year	$43 046.53	1.4142	$35 015.85	1.3653	$8031	0.0489	$164 335	Undominated	-$5587.30	-$3143.92	-$700.53
	6-year	$48 066.67	4.0382	$48 423.61	3.6848	-$357	0.3534	-$1010	Dominant	$18 025.67	$35 694.41	$53 363.14
	10-year	$52 409.01	6.3865	$63 276.70	5.6009	-$10 868	0.7856	-$13 834	Dominant	$50 147.85	$89 428.01	$128 708.17

Abbreviations: ICER, incremental cost-effectiveness ratio; NMB, net monetary benefit; TLIP, transformational lumbar interbody fusion.

a ΔCost = TOPS cost - Control Cost.

b ΔQALY = TOPS QALY - Control QALY.

c ICER = ΔCost / ΔQALY; “Dominant” indicates that TOPS costs less while yielding a higher QALY.

d NMB = ΔQALY *WTP threshold - ΔCost.
